# Advances in Plasmonics and Nanophotonics

**DOI:** 10.3390/nano11113159

**Published:** 2021-11-22

**Authors:** Burak Gerislioglu, Arash Ahmadivand

**Affiliations:** 1 Department of Physics and Astronomy, Rice University, 6100 Main St., Houston, TX 77005, USA; 2 Department of Electrical and Computer Engineering, Rice University, 6100 Main St., Houston, TX 77005, USA; 3 Metamaterial Technologies Inc. (META), Pleasanton, CA 94588, USA

Recent developments in subwavelength localization of light have paved the way of novel research directions in the field of optics, plasmonics, and nanophotonics. Over the past decade, ongoing efforts have shown that one can control the propagation and localization of electromagnetic waves below the incident wavelength toward enhancing light’s electric and magnetic field features. This principle enables complex wavefront manipulation (e.g., amplitude, phase, and polarization modulation) by minimizing possible diffraction effects. The underlying physics of resonant structures, which can easily trap incident light and create high-density concentrations of electromagnetic energy, is the main thrust that drives advances in plasmonics and nanophotonics and brings all-optical communication and data processing one step closer. Lately, researchers have shed light on the remarkable progress in all-dielectric resonant nanophotonics by setting high expectations for novel discoveries and demonstrating many promising applications in imaging, sensing, signal processing, and quantum technologies, indicating that reaching novel horizons for further success of photonics and optics is not solely based on plasmonics. 

This Special Issue includes five articles [1,2,3,4,5] which present some of the challenges and opportunities in the field of plasmonics and nanophotonics. 

Hussien et al. [1] discuss methods to develop a rational understanding of topological electron states and their topological quantum phase transitions for emerging applications in photonics, optoelectronics, and spintronics. The authors demonstrate combined first principles and field-theoretic calculations of the electrodynamic signatures of carriers at characteristic energies at which distinct topological phase transitions occur in Dirac materials. 

Jiang et al. [2] demonstrate a tri-layered metasurface composed of an arrow-type structure sandwiched by a pair of orthogonal gratings. Based on the concept demonstration, anomalous refraction, cylindrical focusing, point focusing, and vortex beams with varied topological charges are investigated. The extracted results show that the proposed metasurface has tremendous potential in developing efficient, broadband, and compact systems for THz wireless communication.

Sun et al. [3] elaborate a method for designing two types of GaN metasurfaces based on the dynamic phase. One of the metasurfaces can generate a focused beam, while the other one can generate a vortex beam. Compared with the focused beam, the vortex beam can carry orbital angular momentum. It is also mentioned that due to the advantages of their high power and high bandwidth, the GaN metasurfaces can be widely used in the industrial communication field and third-generation semiconductor market.

Zhuo et al. [4] propose tunable valley graphene plasmonic metamaterials (VGPMs) for group velocity modulation and light field focusing of surface plasmon polariton waves. The authors further discuss a chirped VGPM waveguide composed of a supercell arrangement of VGPMs with a gradually increasing chemical potential difference. The chirped VGPM waveguide shows prominent potential for nanophotonics systems and for the manipulation of spin–orbit interactions of light, due to its remarkable tunability, backscattering resistance, and low absorption features. 

Iftimie et al. [5] show that intensity-based moments and associated parameters defined in terms of average position, spatial extent, skewness, and kurtosis can adequately capture changes in beam shapes induced by aberrations of a metalens with a hyperbolic phase profile. The extracted numerical results allow the identification of the parameters that are most prone to induce changes in the beam shape for metalenses and indicate that the approach considered here is novel in the study of metalenses, for which ray optics and the associated phase distribution-based methods are the techniques of choice. 

We hope that these articles are of interest to students, researchers, theorizers, and experimentalists working in the field of optics, plasmonics, and nanophotonics, and that exciting ideas will be ignited after reading the contributions made to this Special Issue. 
